# Remifentanil versus fentanyl during cardiac surgery on the incidence of chronic thoracic pain (REFLECT): study protocol for a randomized controlled trial

**DOI:** 10.1186/1745-6215-15-466

**Published:** 2014-11-27

**Authors:** Sjoerd de Hoogd, Sabine JGM Ahlers, Eric PA van Dongen, Dick Tibboel, Albert Dahan, Catherijne AJ Knibbe

**Affiliations:** Department of Clinical Pharmacy, St Antonius Hospital, Koekoeklaan 1, 3435 CM Nieuwegein, The Netherlands; Department of Anesthesiology and Intensive Care, St Antonius Hospital, Koekoeklaan 1, 3435 CM Nieuwegein, The Netherlands; Intensive Care and Department of Pediatric surgery, Erasmus MC - Sophia Children’s Hospital, Wytemaweg 80, 3015 CN Rotterdam, The Netherlands; Department of Anesthesiology, Leiden University Medical Center, Albinusdreef 2, 2333 ZA Leiden, The Netherlands; Division of Pharmacology, Leiden Academic Center for Drug Research, Leiden University, Einsteinweg 55, Leiden, The Netherlands

**Keywords:** Chronic pain, Sternotomy, Remifentanil, Fentanyl, Postoperative pain

## Abstract

**Background:**

Chronic thoracic pain after cardiac surgery is prevalent (11 to 56%) and may affect patients’ physical and mental health status. Despite its favorable pharmacokinetic and pharmacodynamic properties, high doses of remifentanil administered during surgery are reported to cause acute postoperative pain and increased requirements for analgesics. Recently, an association between remifentanil use and the incidence of chronic thoracic pain in the long term was also reported. Our objective is to investigate the influence of the intraoperative remifentanil on chronic postoperative pain in a prospective randomized controlled trial.

**Methods/design:**

In this prospective, randomized, single-blind clinical trial, all patients (N =126) between 18 and 85 years undergoing cardiac surgery via sternotomy receive a continuous infusion of propofol together with intermittent intravenous fentanyl at predetermined times perioperatively. Patients are randomized to receive either an additional continuous infusion of remifentanil (0.15 μg^-1^kgIBW^-1^ min^-1^) or additional fentanyl (200 to 500 μg) as needed during surgery.

The primary end point is the prevalence of chronic thoracic pain 12 months after surgery. Secondary end points include acute postoperative pain; postoperative analgesic use; chronic thoracic pain 3 and 6 months after surgery; quality of life (SF-12) at 3, 6 and 12 months after surgery; work productivity; and use of health care. In addition, thermal detection and pain thresholds are measured preoperatively, 3 days after surgery and 12 months after surgery using quantitative sensory testing (QST). Finally, the influence of several genetic variances on the different outcomes will be measured.

**Discussion:**

Chronic thoracic pain is prevalent after cardiac surgery, and research is needed to minimize the risk of chronic persistent postoperative pain, which is an invalidating, long-term complication of surgery. The objective of this trial is to determine the influence of perioperative remifentanil on long-term pain outcomes for cardiac patients in a prospective randomized trial. The results may be used to optimize perioperative analgesia techniques and, thereby, improve quality of life after cardiac surgery.

**Trial registration:**

Clinicaltrials.gov NCT02031016 on 13 December 2013.

## Background

Remifentanil is a pain-relieving drug frequently used during surgery due to its favorable pharmacokinetic and pharmacodynamic properties. It is characterized by rapid onset, predictable rapid recovery profile and dosing reliability [1]. Its use is associated with a shorter length of hospital stay and duration of mechanical ventilation after cardiac surgery and a cardioprotective effect in coronary artery bypass graft (CABG) surgery patients [2, 3]. On the other hand, high doses of remifentanil administered during surgery have been reported to cause acute postoperative pain and opioid-induced hyperalgesia [4]. Acute postoperative pain, in turn, is a major risk factor for the development of chronic pain [5–7]. Studies report incidences of chronic thoracic pain after cardiac surgery via sternotomy varying from 11% to 56%, depending on the definition and the study population [8–12]. These patients reported significantly lower physical and mental health status compared to patients without chronic thoracic pain [8, 11, 13, 14].

Little is known about a possible association between the intraoperative use of remifentanil and the development of chronic pain. A dose-dependent relationship was shown in 90 cardiac patients one year after surgery [15]. Also a randomized study designed to evaluate allodynia after thoracotomy and the occurrence of chronic thoracic pain suggested that there might be an association between the use of high dose remifentanil and increased prevalence of chronic thoracic pain [16]. More recently, a retrospective study found that the combination of remifentanil and sevoflurane was less favorable in terms of chronic pain compared to a propofol and remifentanil combination. Patients with chronic pain had received a significantly higher dose of remifentanil, but significance was not reached in multivariate analysis [17].

In our hospital, about 60% of cardiac surgery patients receive remifentanil next to fentanyl intraoperatively, depending on the anesthetist’s preference [15]. We wonder whether the possible development of hyperalgesia and chronic thoracic pain, with the negative impact on quality of life and cost efficacy, carries the risk of overcoming the advantages of remifentanil.

So far, however, no prospective randomized controlled trials designed to evaluate the influence of intraoperative remifentanil on the incidence of chronic thoracic pain are available. The current prospective randomized trial is designed to investigate the influence of perioperative additional remifentanil or additional fentanyl on the development of chronic thoracic pain at 3, 6 and 12 months after surgery. In addition, changes in thermal detection thresholds and pain thresholds and the influence of genetic variances will be investigated.

## Methods/design

### Study design

**Figure 1:**
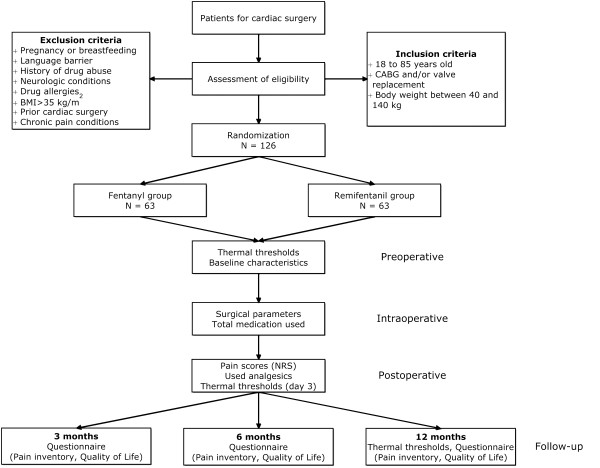
**Flowchart of study outline.**

This study is a prospective, randomized, single-blind clinical trial carried out in the St. Antonius Hospital, Nieuwegein, the Netherlands (Figure 1). The study population consists of adult cardiac patients undergoing elective coronary artery bypass (CABG) surgery and/or valve replacement surgery via sternotomy. Patients are blinded for treatment and are randomly assigned to the remifentanil or fentanyl group.

The study was approved by the local Ethics Committee of this hospital (Verenigde Commissies Mensgebonden Onderzoek (VCMO) R13.013). The study was registered on the Clinical Trials register on 13 December 2013 (ClinicalTrials.gov number NCT02031016). The research coordinator will obtain written informed consent from each participant.

### Eligibility

The following inclusion criteria are being applied: (1) patients undergoing cardiac surgery via sternotomy (a CABG and/or valve replacement); and (2) age between 18 and 85 years; (3) weight between 45 and 140 kg. Exclusion criteria are: (1) pregnancy or breastfeeding; (2) language barrier; (3) history of drug abuse; (4) neurologic condition such as peripheral neuropathy and fibromyalgia; (5) known remifentanil, fentanyl, morphine or paracetamol allergy; (6) a body mass index (BMI) above 35 kg/m^2^ (7) prior cardiac surgery (re-operations); and (8) patients with chronic pain conditions.

### Interventions

#### Intraoperative analgesic protocol

Anesthesia will be induced with midazolam (2.5 to 5.0 mg) followed by a propofol bolus (1 to 2 mg/kg), fentanyl (250 to 500 ug) and pancuronium (0.05-2 mg/kg). After tracheal intubation, patients will be ventilated to normocapnia with oxygen enriched air (50 to 100% oxygen). No nitrous oxide will be used. Sevoflurane will be used as needed. Both groups receive a continuous infusion of propofol (starting dose 200 to 300 mg/hour) and intermittent intravenous fentanyl at predetermined times (that is, before incision, at sternotomy, at aorta cannulation and at opening of the pericardium).

Patients in the remifentanil group will receive a continuous infusion of remifentanil (starting infusion dose 0.15 ug/kg ideal body weight (IBW)/min) on top of this propofol-fentanyl anesthetic regimen. Patients in the fentanyl group will receive additional boluses of fentanyl 200 to 500 μg as needed instead of the remifentanil infusion.

Patients in both groups will receive 5 to 10 mg of intravenous morphine 30 minutes before the anticipated end of surgery. After surgery, patients will either go to the Intensive Care Unit (ICU) or to the Post-Anesthesia Care Unit (PACU).

#### Quantitative sensory testing

One day before surgery, three days after surgery and one year after surgery quantitative sensory testing (QST) will be performed. Cold and warm detection thresholds and pain thresholds will be measured, using the ‘method of limits’ with the Thermal Sensory Analyzer (TSA) II 2001 (Medoc Advanced Medical Systems, U.S.) [18, 19]. The threshold values will be corrected for age, gender and reaction time. Before the first test session, patients will be asked to practice testing at least twice. A test-retest variability of less than 20% is required before formal pain testing begins. The TSA II has been used extensively to determine warm/cold detection limits and warm/cold pain thresholds.

#### Postoperative treatment

Patients in both groups will receive the same postoperative treatment. In both the ICU and the PACU, a pain management protocol is in place as part of standard care, consisting of a continuous infusion of morphine (starting dose of 2 mg/hour), which is adapted depending on Numerical Rating Scale (NRS) pain scores, as well as paracetamol four times a day (1 g oral/intravenous) [20]. Any perioperative fentanyl or remifentanil is discontinued at arrival at the ICU or PACU. NRS scores are assessed three times a day and administered medication is registered as part of standard care. On the general postoperative wards, the pain protocol consists of 2.5 to 10 mg morphine (oral/intravenous) on demand and paracetamol four times a day (1 g oral).

#### Follow-up

After discharge from the hospital, patients will be asked to complete questionnaires 3, 6 and 12 months after surgery. This questionnaire contains questions about pain (perception, location, intensity) based on the Brief Pain Inventory [21] and was described previously [5]. In addition, quality of life (QoL) is measured with the short form (SF)-12 health status instrument, and work productivity and use of health care resources are measured. One year after cardiac surgery, pain thresholds are measured, using the same QST protocol.

### Primary end point and secondary end points

The primary end point is the percentage of patients with chronic thoracic pain (NRS >0) one year after cardiac surgery.

Secondary end points are mean daily acute postoperative NRS scores (0 to 10) and analgesic consumption until discharge from the hospital. Also chronic pain (NRS), Quality of Life, analgesic consumption, work productivity, and use of health care 3, 6 and 12 months after surgery are assessed. In addition, warm/cold detection and pain thresholds (absolute and relative to preoperative values (baseline)) using quantitative sensory testing (QST) are considered. The length of ICU, PACU and hospital stay will be calculated.

In addition to primary and secondary endpoints, an exploratory screening of different genes in this population will be investigated. The possible influence of different genetic variances that are involved in pain sensitivity (for example, GTP-cyclohydrolase 1 (GCH-1), WDFY family member 4 (WDFY4), Zinc Finger gene Family (ZNF), Melanocortin 1 Receptor (MC1R)) and the pharmacokinetics and pharmacodynamics of opiates (for example, glucuronosyl transferase (UGT), Multidrug Resistance-associated Protein (MRP), mu-opioid receptor gene 1 (OPRM1), Catechol-O-methyltransferase (COMT)) will be explored.

### Data analysis

Statistical analyses will be done with The SPSS statistical package (version 22.0 for Windows; SPSS, Chicago, IL). Patient demographics, baseline characteristics and clinical observations are compared between patients receiving remifentanil versus fentanyl during cardiac surgery. Nonparametric data will be expressed as median (range) and analyzed by chi-square. Parametric data will be expressed as mean ± SD and analyzed by Student’s t-tests or ranks tests.

The effect of intraoperative use of remifentanil on chronic pain one year after cardiac surgery is analyzed using univariate logistic regression analysis. If baseline characteristics are not balanced, multivariable techniques will be applied. To evaluate the effect of remifentanil versus fentanyl during cardiac surgery on pain thresholds, paired t-tests will be used.

To estimate the effect of the genotype on the outcome variables, each gene is examined to determine the appropriate model. The gene variants will be coded based on the observed distribution. The outcome parameters are compared between genotypes by a linear mixed model analysis based on the maximum likelihood ratio with the patient genotype status as fixed factors and the time point of outcome parameters as repeated measurement.

### Sample size calculations

In a previous study of 90 patients on chronic thoracic pain after cardiac surgery, 15 of the 52 patients who received remifentanil developed chronic thoracic pain (29%) versus 3 of the 38 patients who did not receive remifentanil (8%); resulting in an odds ratio of 4.7 [15].

Rounding numbers, the sample size calculation is made on the assumption that in this prospective study, approximately 30% of the patients receiving remifentanil will develop chronic thoracic pain and that approximately 10% of the patients receiving fentanyl will develop chronic thoracic pain.

The sample size is calculated with a power of 0.80 and an alpha of 0.05; two sided. A total number of 117 patients are needed. According to previous reports, mortality thirty days after cardiac surgery is approximately 2 to 13.3% [22, 23]. In a previous study, 8.4% died within one year after surgery [15]. Therefore, the total number of patients needed in this trial is 117 * 1.08 = 126; 63 patients in each arm.

## Discussion

This is the first randomized trial that prospectively evaluates the influence of intraoperative remifentanil on the incidence of chronic thoracic pain. Studies in healthy volunteers indicate that remifentanil increases the occurrence of secondary hyperalgesia in experimental pain models [24–28]. Other studies have described higher pain levels or analgesic requirements in the acute phase after surgery upon intraoperative use of remifentanil [4, 29, 30]. The clinical long-term relevance of these mostly short-term increases in pain scores, analgesic requirements or secondary hyperalgesia is unknown.

The putative biological mechanism by which remifentanil would cause chronic pain is unclear. Opioid-induced hyperalgesia is well described in animal studies but the occurrence in patients is still under debate [31]. Animal studies suggest that remifentanil influences the N-methyl-D-aspartate (NDMA) currents by affecting opioid receptors [32, 33]. Modulation of these NDMA currents could lead to central sensitization and consequently possibly to chronic postoperative pain.

Ideally, the study design should be double blind and contain no other opioid besides remifentanil. As a study arm without opioids during surgery is obviously unethical, fentanyl was selected for the other arm. The current design, where intermittent fentanyl at predetermined times is combined with continuous propofol as the basis for standardized Total Intravenous Anesthesia (TIVA) in both arms, is based on the design of a previous study investigating risk factors for chronic thoracic pain in our hospital [15]. With one arm randomized to an additional remifentanil infusion and one arm randomized to additional intermittent fentanyl as needed, these two study groups can both be considered the standard of care in our hospital. Given the familiarity with the two study arms, we do not expect unintentional effects from the nonblinding of anesthesiologists and ward nurses. It is emphasized that the patient is kept blinded for the treatment group, which is of particular relevance since it is the patient who determines the primary endpoint, that is, chronic thoracic pain one year after surgery. Another potential design that was considered was a group receiving remifentanil only with another group receiving fentanyl only. While the remifentanil-only group, in particular, cannot be considered a standard of care treatment in our hospital, we underline that when the current design was used in a previous nonrandomized study, a difference in the prevalence of chronic thoracic pain was detected [15].

In the current study, all patients will be evaluated for their sensory detection and pain thresholds using QST preoperatively, 3 days and 1 year after surgery. This randomized trial in which chronic thoracic pain after cardiac surgery is studied in the remifentanil and control arm is not only an opportunity to investigate the influence of remifentanil on sensory thresholds, but also to explore the influence of cardiac surgery and chronic pain on sensory modalities. Ideally, for this purpose, a pain battery with more than one stimulus (for example, electricity and pressure) should be used; however, in our opinion, use of a large number of pain thresholds measurements is not feasible in patients prior to and 3 days after invasive cardiac surgery.

The results of this randomized trial may be used to optimize intraoperative analgesia techniques and thereby improve the quality of life of patients after cardiac surgery.

### Trial status

The trial is currently enrolling patients. To date, 90% of the patients have been enrolled in the study. The expected average enrollment rate is three to five patients every week. Data collection will stop one year after the last patient is included.

## Abbreviations

BMI: body mass index
BPI: Brief Pain Inventory
CABG: coronary artery bypass graft
COMT: catechol-O-methyltransferase
GCH-1: GTP-cyclohydrolase 1
IBW: ideal body weight
ICU: intensive care unit
LBW: lean body weight
MC1R: melanocortin 1 receptor
MRP: multidrug resistance-associated protein
NMDA: N-methyl-D-aspartate
NRS: Numerical Rating Scale
OPRM1: mu-opioid receptor gene 1
PACU: post-anesthesia care unit
QoL: quality of life
QST: quantitative sensory testing
SF-12: Short Form-12
TSA: thermal sensory analyzer
UGT: glucuronosyl transferase
WDFY4: WDFY family member 4
ZNF: zinc finger gene family.
